# Effect of periodontal treatment on diabetes-related healthcare costs: a retrospective study

**DOI:** 10.1136/bmjdrc-2020-001666

**Published:** 2020-10-23

**Authors:** Kirsten P J Smits, Stefan Listl, Adelina S Plachokova, Onno Van der Galien, Olivier Kalmus

**Affiliations:** 1 Department of Dentistry - Quality and Safety of Oral Healthcare, Radboud University Medical Center, Radboud Institute for Health Sciences, Nijmegen, The Netherlands; 2 Department of Conservative Dentistry, Section for Translation Health Economics, Heidelberg University, Heidelberg, Baden-Württemberg, Germany; 3 Department of Dentistry - Implantology and Periodontology, Radboud University Medical Center, Radboud Institute for Health Sciences, Nijmegen, The Netherlands; 4 Vision and Innovation, Achmea, Zeist, The Netherlands

**Keywords:** diabetes mellitus, type 2, periodontal diseases, health care costs, cohort studies

## Abstract

**Introduction:**

Periodontitis has been considered a sixth complication of diabetes. The aim of this study was to assess the impact of periodontal treatment on diabetes-related healthcare costs in patients with diabetes.

**Research design and methods:**

A retrospective analysis was done, exploiting unique and large-scale claims data of a Dutch health insurance company. Data were extracted for a cohort of adults who had been continuously insured with additional dental coverage for the years 2012–2018. Individuals with at least one diabetes-related treatment claim in 2012 were included for analysis. A series of panel data regression models with patient-level fixed effects were estimated to assess the impact of periodontal treatment on diabetes-related healthcare costs.

**Results:**

A total of 41 598 individuals with diabetes (age range 18–100 years; 45.7% female) were included in the final analyses. The median diabetes-related healthcare costs per patient in 2012 were €38.45 per quarter (IQR €11.52–€263.14), including diagnoses, treatment, medication and hospitalization costs. The fixed effect models showed €12.03 (95% CI −€15.77 to −€8.29) lower diabetes-related healthcare costs per quarter of a year following periodontal treatment compared with no periodontal treatment.

**Conclusions:**

Periodontitis, a possible complication of diabetes, should receive appropriate attention in diabetes management. The findings of this study provide corroborative evidence for reduced economic burdens due to periodontal treatment in patients with diabetes.

**Significance of this study**

**What is already known about this subject?**

- Type 2 diabetes and periodontitis have a bi-directional relationship.
- Treatment of periodontitis may lead to lower hemoglobin A1c values in individuals with type 2 diabetes.

**What are the new findings?**

- Periodontal treatment may lead to a €12 reduction of diabetes-related healthcare costs per patient per quarter of the year.
- More advanced periodontal treatment may lead to higher reductions in diabetes-related healthcare costs.
- Mainly individuals on insulin therapy may benefit financially from periodontal treatment.

**How might these results change the focus of research or clinical practice?**

- These findings underscore the potential importance of periodontal treatment in individuals with diabetes.
- They may support initiatives to promote additional attention to the periodontal status of individuals with diabetes and to raise more awareness for the benefits of periodontal treatment.

## Introduction

The incidence, prevalence, progression and severity of periodontitis have been shown to be higher in individuals with diabetes.^1^ The prevalence of periodontitis in individuals with diabetes is 2 to 3 times higher than in individuals without diabetes.^2 3^ Moreover, diabetes and periodontitis appear to have a bi-directional relationship.^4 5^ Individuals with diabetes are more likely to have more severe periodontitis. Furthermore, individuals with diabetes who also suffer from periodontitis, exhibit more difficulties to stabilize metabolic control, and they develop other diabetes complications more frequently.^1^ Partly because of this, it was previously suggested to consider periodontitis the sixth complication of diabetes.^6^

Reviews of intervention studies show moderately decreasing effect on HbA1c,^5 7 8^ although others found contradictory results.^9 10^ This inconsistency may be explained by differences in the investigated periodontal treatment and definition of periodontitis in the primary studies.^7 8^ If periodontal treatment indeed lowers HbA1c levels, this could lead to better glycemic control and fewer diabetes-related complications. In turn, this could lead to lower healthcare utilization, such as hospitalizations or emergency room visits, and thereby a reduction in the healthcare costs.

The existing evidence on the impact of periodontal treatment on healthcare costs remains unclear. Two cost-effectiveness studies using simulated data suggest that non-surgical periodontal treatment and lifetime maintenance treatment may be cost saving, due to health benefits attributable to HbA1c reductions in patients with type 2 diabetes.^11 12^ A recent review on periodontal treatment and impact on healthcare costs in patients with diabetes identified only three published studies, all from the USA.^13^ One study suggested that overall healthcare costs increased by US$19 per person per month,^14^ whereas the other two studies showed that overall healthcare costs decreased by US$75^15^ and US$237^16^ following periodontal treatment. Uncertainty remains on the impact of periodontal treatment on diabetes costs, and there is a paucity of empirical evidence from outside the USA, certainly also for the Netherlands.

In the Netherlands, as well as the rest of the world, the prevalence of diabetes has been increasing for years.^17^ As a consequence, the diabetes-related healthcare costs have been increasing as well, from €807 million in 2005 to €1.55 billion in 2015.^18^ If the suggested benefit of periodontal treatment on diabetes-related outcomes is true, this may be an efficient way to improve the health of patients with diabetes, and reduce the healthcare costs. We therefore aimed to assess whether periodontal treatment has an influence on diabetes-related healthcare costs in Dutch patients with diabetes. We hypothesized that people with diabetes will have lower diabetes-related healthcare costs following periodontal treatment.

## Methods

Retrospective analyses were undertaken, exploiting anonymized claims-level administrative data spanning a 7-year period from 2012 to 2018 from the Achmea Health Database. The ‘Achmea Health Database’ is the private database created by the insurance company Achmea for research purposes.

### Data source and population

Claims data were obtained from individuals (≥18 years) who were continuously insured with additional dental coverage from 2012 to 2018 at Achmea. Achmea provides a wide range of health, life and non-life insurances. In the Netherlands, everyone is obliged to have healthcare insurance. Dental healthcare costs are not part of the standard coverage and should be additionally insured to be reimbursed. From all the individuals, all reimbursement costs and number of claims for periodontal treatment and diabetes-related healthcare were extracted per quarter of a year for the period of 2012–2018. Additionally, data on age (in 5-year categories), gender and socioeconomic status (SES) were extracted. Due to privacy regulations, these personal characteristics were only extracted for 2012 and were therefore fixed variables for the remainder of the follow-up. All individuals who received at least one reimbursement for diabetes-related healthcare in 2012 were included in the study population. Thereafter, all individuals who did not receive any additional reimbursements for diabetes-related healthcare in the remainder of the study period (2013–2018), or without reimbursement for glucose-lowering drugs in 2012 were excluded from analysis. We assumed that these excluded individuals do not actually have type 1 or 2 diabetes. As such, they did not fall within the scope of this research.

### Exposure of interest

We assumed that patients had periodontitis if they received any periodontal treatment. In the Netherlands, the Dutch Periodontal Screening Index (DPSI) is used to screen for periodontitis. DPSI is an internationally validated index, and individuals with DPSI scores 0, 1 and 2 are classified as having no periodontitis (category A), those with DPSI score 3− as having mild periodontitis (category B; periodontal pocket depths of 4–5 mm) and subjects with DPSI scores 3+ and 4 as having severe periodontitis (category C; periodontal pockets depths of ≥6 mm in combination with clinical attachment loss).^19^ Patients with DPSI category B and C proceed further to comprehensive periodontal examination with periodontal chart and radiographs, and are eligible for periodontal treatment. For reimbursement, periodontal treatment codes are used (see online supplemental etable 1). These codes are based on the comprehensive periodontal examination and stand for initial periodontal therapy, periodontal surgery, supportive/maintenance periodontal therapy with different durations of the follow-up sessions (ie, short, normal and long), and treatment of periodontal complications, such as periodontal abscess. For purposes of secondary analyses, the periodontal treatment codes were classified into intermediate and advanced periodontal treatment based on the level of comprehensiveness of the treatment itself. Intermediate periodontal treatment was defined as any reimbursement for initial periodontal treatment, short term and normal follow-up sessions. Advanced periodontal treatment was defined as any reimbursement for periodontal surgery, long follow-up sessions and treatment of periodontal complications.

10.1136/bmjdrc-2020-001666.supp1 Supplementary data

### Outcome of interest

Diabetes-related healthcare costs were based on reimbursement codes related to diabetes diagnosis, treatment, medication and hospitalizations (see online supplemental etable 2). Hospitalizations included codes related to inpatient stays, outpatient visits related to diabetes and diabetic foot or eye complications. For secondary analyses, three mutually exclusive categories were created based on diabetes medication use in 2012: only metformin; other oral blood glucose-lowering drugs (which may include use of metformin in combination with other drugs) and insulin. These groups are based on the medication treatment steps recommended in Dutch diabetes guidelines.^20^

### Statistical analysis

Descriptive statistics were used to describe the population. Frequencies and percentages were reported for categorical variables, and median and IQRs for the variables regarding healthcare costs. Wilcoxon rank-sum test and χ^2^ test were used to assess differences between people with and without any reimbursement for periodontal treatment in 2012–2018.

To assess the possible association between periodontal treatment and total diabetes-related healthcare costs, a regression model was performed. To control for unobserved (time-invariant) individual characteristics, we estimated a panel regression fixed effects model. Computationally, this was implemented via the Stata command ‘xtreg’. This model is based on individual, longitudinal data.^21^ Periodontal treatment was the independent variable. Once a person received any reimbursement for periodontal treatment, the person was classified as receiving periodontal treatment from the next quarter of a year on for the remainder of the follow-up. The primary outcome was total diabetes-related healthcare cost per quarter of a year. In secondary analyses, periodontal treatment was classified in intermediate and advanced treatment to assess how this affected the total diabetes-related healthcare costs. P values <0.05 were considered to be statistically significant. All analyses were performed in Stata/SE V.14.2.

### Sensitivity analyses

Several sensitivity analyses were performed. First, models with secondary outcome variables diabetes medication-related healthcare costs and non-medication-related healthcare costs were performed to assess if some costs were more affected than others by periodontal treatment. Second, the regression models were performed separately for each diabetes medication category to assess whether some patient groups were more affected than others. Third, for the fixed effect regression models it was determined whether the time lag between receiving the first periodontal treatment reimbursement and the coding of this variable had any influence. For this, the time lag was expanded to two quarters (equals 6 months) and four quarters (equals 1 year). Finally, the primary fixed effect models were repeated for subsamples of the population regarding age (≤65 years vs >65 years), gender (male vs female) and SES (very low-low vs neutral-high-very high).

## Results

**Figure 1:**
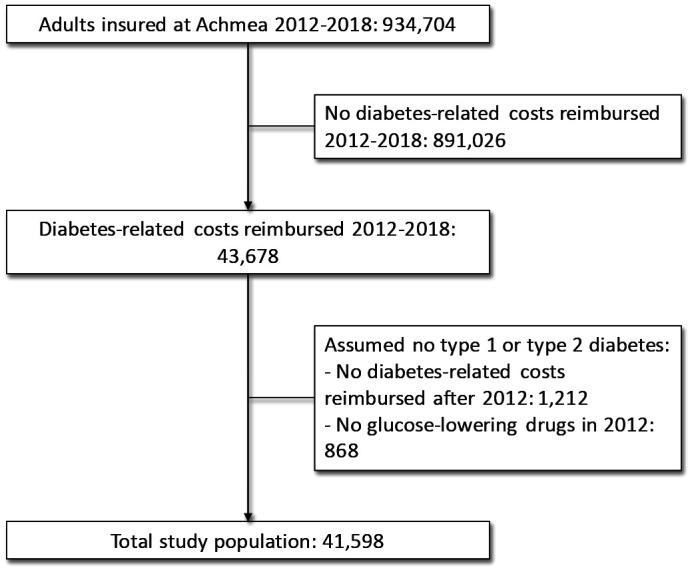
Flow chart of included and excluded people from the original sample.

In total, 934 704 adults were observed for the whole period of 2012–2018. Of these, 43 678 received a reimbursement for diabetes-related healthcare costs. After exclusion of 1212 individuals with no diabetes-related claims in the follow-up period of 2013–2018 and 868 individuals without claims for glucose-lowering drugs in 2012, a study population of 41 598 individuals remained (figure 1). Most individuals were in the 50–75 years age categories in 2012 (70%), and 54% were men (table 1). Median total diabetes-related healthcare costs per person ranged from €38.45 to €59.17 per quarter between 2012 and 2018, and median periodontal treatment costs per person ranged from €29.06 to €31.94 per quarter between 2012 and 2018. Descriptive analysis between people with reimbursement for periodontal treatment in 2012–2018 and those without showed significant differences for gender, age, SES, diabetes medication categories in 2012 and total diabetes-related healthcare costs for all years between 2012 and 2016.

**Table 1:** Baseline characteristics of study population

	Total study population (n=41 598)	People with periodontal treatment in 2012–2018 (n=8188)	People without periodontal treatment in 2012–2018 (n=33 410)	χ^2^ p value
Gender, No. (%)				
Men	22 605 (54.3)	4721 (57.7)	17 884 (53.5)	<0.001
Women	18 992 (45.7)	3467 (42.3)	15 525 (46.5)	
Unknown	1 (0.0)	0 (0.0)	1 (0.0)	
Age, No. (%), years				
18–25	475 (1.1)	36 (0.4)	439 (1.3)	<0.001
25–30	460 (1.1)	55 (0.7)	405 (1.2)	
30–35	666 (1.6)	101 (1.2)	565 (1.7)	
35–40	1139 (2.7)	212 (2.6)	927 (2.8)	
40–45	2198 (5.3)	427 (5.2)	1771 (5.3)	
45–50	3513 (8.5)	778 (9.5)	2735 (8.2)	
50–55	4867 (11.7)	1152 (14.1)	3715 (11.1)	
55–60	6120 (14.7)	1356 (16.6)	4764 (14.3)	
60–65	6977 (16.8)	1518 (18.5)	5459 (16.3)	
65–70	6752 (16.2)	1323 (16.2)	5429 (16.3)	
70–75	4357 (10.5)	703 (8.6)	3654 (10.9)	
75–80	2621 (6.3)	365 (4.5)	2256 (6.8)	
80–85	1161 (2.8)	136 (1.7)	1025 (3.1)	
85–90	263 (0.6)	22 (0.3)	241 (0.7)	
90–95	28 (0.1)	3 (0.0)	25 (0.1)	
95–100	1 (0.0)	1 (0.0)	0 (0.0)	
SES, No. (%)				
Very low	9362 (22.5)	2141 (26.2)	7648 (21.6)	<0.001
Low	10 256 (24.7)	2249 (27.5)	7148 (24.0)	
Moderate	3976 (9.6)	929 (11.4)	3047 (9.1)	
High	8516 (20.5)	1368 (16.7)	8007 (21.4)	
Very high	9070 (21.8)	1422 (17.4)	7221 (22.9)	
Unknown	418 (1.0)	79 (1.0)	339 (1.0)	
Diabetes medication in 2012, No. (%)				
Only metformin	14 534 (34.9)	2985 (36.5)	11 549 (34.6)	0.001
Other oral DM medication†	13 254 (31.9)	2605 (31.8)	10 649 (31.9)	
Insulin	13 810 (33.2)	2598 (31.7)	11 212 (33.6)	
Diabetes-related healthcare costs per patient per quarter, median (IQR), €				
2012	38.45 (11.52–263.14)	33.14 (10.90–257.83)	40.22 (11.67–264.72)	0.003
2013	41.00 (11.64–267.60)	34.37 (11.13–267.79)	42.48 (11.78–267.50)	0.023
2014	44.42 (12.76–262.91)	38.17 (12.29–263.01)	46.04 (12.90–262.87)	0.016
2015	47.11 (13.10–228.39)	40.87 (12.36–227.87)	48.53 (13.27–228.44)	0.009
2016	59.17 (14.80–248.21)	50.47 (14.25–246.40)	61.57 (14.93–248.34)	0.040
2017	39.29 (4.88–225.60)	34.09 (4.86–230.04)	40.41 (4.89–224.56)	0.597
2018	46.58 (5.04–223.62)	40.08 (5.09–226.32)	47.89 (5.03–223.01)	0.773
Periodontal treatment costs per patient per quarter, median (IQR), €*				
2012	29.74 (17.00–41.09)	29.74 (17.00–41.09)	0 (0.0–0.0)	<0.001
2013	29.06 (15.39–41.04)	29.06 (15.39–41.04)	0 (0.0–0.0)	<0.001
2014	31.94 (16.86–42.97)	31.94 (16.86–42.97)	0 (0.0–0.0)	<0.001
2015	30.83 (16.25–43.60)	30.83 (16.25–43.60)	0 (0.0–0.0)	<0.001
2016	30.13 (20.02–45.19)	30.13 (20.02–45.19)	0 (0.0–0.0)	<0.001
2017	30.66 (20.37–45.98)	30.66 (20.37–45.98)	0 (0.0–0.0)	<0.001
2018	31.50 (20.93–47.24)	31.50 (20.93–47.24)	0 (0.0–0.0)	<0.001

*Periodontal treatment costs only for those with periodontal treatment.

†May include metformin in combination with other oral blood glucose-lowering drugs.

DM, diabetes mellitus; NA, not applicable; SES, socioeconomic status.

**Figure 2:**
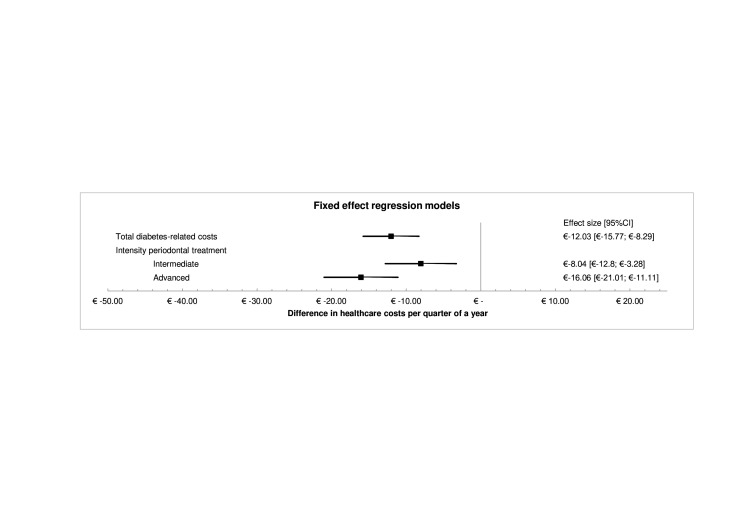
Fixed effects regression models for periodontal treatment and total diabetes-related costs, including secondary analyses for intensity of periodontal treatment.

The fixed effects models showed a significant reduction in total diabetes-related costs of −€12.03 (95% CI −€15.77 to −€8.29) (p<0.001) per quarter year in patients after periodontal treatment compared with no periodontal treatment (table 2, figure 2). Secondary analyses showed larger reductions for the advanced periodontal treatment group compared with the intermediate periodontal treatment group.

**Table 2:** Fixed effect regression analysis

Model	Coefficient	95% CI	P value
Any periodontal treatment	−€12.03	−€15.77 to −€8.29	<0.001
Intensity of periodontal treatment			
Intermediate periodontal treatment	−€8.04	−€12.80 to −€3.28	0.001
Advanced periodontal treatment	−€16.06	−€21.01 to −€11.11	<0.001

The first sensitivity analysis showed increased medication-related healthcare costs and decreased non-medication-related healthcare costs after periodontal treatment, respectively €4.92 (95% CI €3.93 to €5.91) (p<0.001) and −€16.95 (95% CI −€20.53 to −€13.37) (p<0.001) per patient per quarter. The second sensitivity analysis showed significantly increased total diabetes healthcare costs for the ‘only metformin’ (€7.38 (95% CI €4.38 to €10.37), p<0.001) and the ‘other blood glucose-lowering drugs’ group (€13.56 (95% CI €8.51 to €18.61), p<0.001), whereas significantly decreased costs were found for the ‘insulin’ group (−€58.09 (95% CI −€67.83 to −€48.35), p<0.001). Further sensitivity analysis showed that different time lags between receiving the first periodontal treatment and coding of this variable did not lead to relevant changes of the association. More details on the results of the sensitivity analyses and the analyses in subsamples of the study population can be found in online supplemental etable 3 and etable 4.

## Discussion

The findings of this study suggest a significant reduction of around −€12 in the quarterly diabetes-related healthcare costs per person following periodontal treatment in individuals with assumed diabetes. Furthermore, the findings indicate that more advanced periodontitis treatment may result in higher benefits, the benefits are mainly attributable to non-medication-related healthcare costs, and that different patient groups may benefit differently from periodontal treatment.

The findings presented in this study are in line with two previous claims data studies showing reductions in (diabetes-related) healthcare costs for patients with diabetes after receiving periodontal treatment,^15 16^ although our results show smaller reductions. This may be explained because of differences in study design or context. The study by Nasseh *et al*^15^ focused on patients with newly diagnosed diabetes, and the reported diabetes healthcare costs were much higher than observed in the current study. The study by Jeffcoat *et al*^16^ defined periodontal treatment as receiving more than three periodontal treatments compared with three or less periodontal treatments as the control group. Both studies, however, suggest lower diabetes-related healthcare costs after periodontal treatment in patients with diabetes. This was confirmed by a recent cost-effectiveness study showing that providing periodontal treatment to patients with type 2 diabetes would be cost saving.^12^

Besides the overall finding that periodontal treatment reduces overall diabetes-related healthcare costs, these findings suggest that individuals receiving advanced periodontal treatment benefit more than individuals receiving intermediate periodontal treatment. The dose-response relation between periodontitis and diabetes may play a role here.^22^ Possibly people receiving advanced periodontal treatment have more severe periodontitis and may benefit more from this treatment. More severe periodontitis may have a greater impact on diabetes status as well.

The overall reduction of diabetes-related costs through periodontal treatment seems to be largely attributable to patients receiving insulin but not to patients receiving metformin or other oral blood glucose-lowering drugs. It should be noted that the costs of metformin became lower in 2017 and 2018, which partly explains the lower diabetes costs overall in these years. According to Dutch type 2 diabetes guidelines, insulin is the third and final step in pharmacotherapy after metformin and sulfonylurea derivatives.^20^ It may be that the effect of periodontal treatment is dependent on diabetes severity, that is patients with more severe diabetes (who receive insulin medication) benefit more from periodontal treatment compared with those with less severe diabetes (who receive metformin or other oral blood glucose-lowering drugs). However, it should be mentioned that the diabetes medication groups were based on medication reimbursement claims in 2012. Patients can switch from medication regime, which was not controlled for in this study. The observed effect of periodontal treatment resulting in increased diabetes-related costs for patients receiving metformin or other oral blood glucose-lowering drugs may reflect adaptations in the diabetes management regime.

Besides lowering HbA1c levels and financial benefits of periodontal treatment in individuals with diabetes,^5 7 8^ periodontal treatment could also have additional benefits. Severe periodontitis is linked to a higher risk of diabetes complications,^23^ and it might be hypothesized that treatment of periodontitis and improving the periodontal status may lead to a lower risk of these complications. A retrospective study in Taiwan found lower rates of cardiovascular disease among individuals with diabetes and advanced periodontal treatment compared with those with non-advanced periodontal treatment.^24^ In addition, periodontal treatment in patients with diabetes has been associated with improved quality of life and higher diabetes treatment satisfaction.^25 26^ Periodontal treatment in individuals with diabetes may therefore be beneficial on multiple outcomes.

The findings of this study support the integration of medical and dental healthcare, especially in patients with diabetes. The Dutch guidelines for type 2 diabetes already suggest short oral health checks during the yearly diabetes visit and recommends to advice patients with diabetes to see the dentist twice a year.^20^ The current findings contribute to the evidence for these statements and might even suggest to raise more awareness for periodontitis as the sixth complication of diabetes. Vice versa, the dentist could play an important role by assessing diabetes risk in high-risk patients such as those with periodontitis. Easy screening tools for diabetes exist, such as the Finnish Diabetes Risk Score,^27^ and could be included in standard patient evaluations.^28^ Possibly, the development and advancement of electronic decision support systems might provide unique and novel opportunities for integrated management of patients with diabetes and periodontitis.^29^

The present study has some strong features to be mentioned. First, it used a large database including all insured individuals at one of the largest health insurers in the Netherlands. In total, almost 1 million individuals were included in the original sample, which is almost 6% of the total Dutch inhabitants. Then, we included all individuals with assumed diabetes in 2012, giving us a sample of over 40 000 individuals with a follow-up period of 7 years. For these 7 years, extensive data were available regarding the number of claims and the reimbursements for periodontitis-related and diabetes-related healthcare costs. We were able to perform fixed effect models, which corrected for confounding variables and accounted for the longitudinal data.^21^

Despite the extensiveness of the data, the nature of the data was claims data. As such, we had no information on the outcome of clinical measures, oral hygiene or quality of life. However, claims data is usually very well recorded, and therefore the influence of information bias often observed for retrospective studies is limited. Additionally, we had limited access to descriptive variables due to privacy regulations. By using fixed effects models, we adjusted for unobserved heterogeneity to the maximum extent possible with the data at hand. Furthermore, because dental care is not part of the social health insurance package for adults, dental care utilization is only reimbursed and registered via the health insurers for individuals who have opted for additional private dental coverage. Therefore, our initial sample of 1 million individuals included only individuals with additional dental coverage. While this may have affected the external validity of our study through selection bias (ie, generalizability for the general Dutch population) to some extent, our data source still warrants internal validity for a study sample which represents about 1 million individuals. The generalizability of the findings to other countries however, can be seen as limited. Diabetes-related healthcare costs may differ substantially between countries, although earlier studies in the USA found higher financial benefits after periodontal treatment in patients with diabetes.^15 16^

In addition, the dental coverage has a maximum reimbursement ceiling per year. The maximum depends on the type of dental coverage and ranges from €250 to €1250 per year. People exceeding this boundary must pay the additional costs out-of-pocket. Therefore, this study may underestimate the claims for periodontitis. However, individuals who received periodontal treatment once were included in the treatment group always thereafter. As such, we did not distinguish between individuals with just a few periodontal treatments compared with those with many periodontal treatments, and presumably more severe periodontitis. Instead, we distinguished between intermediate and advanced periodontal treatment. Since the clinical status of the individuals included in this study is unknown, it was uncertain whether people included in the non-treated group experienced periodontitis. It can be assumed that a large proportion did not have periodontitis and would likely not benefit from cost savings due to periodontal treatment. Therefore, our findings may be considered to provide lower bound estimates of actual effect sizes.

To overcome some of the limitations present in the current study, a longitudinal, observational study may be performed to assess the long-term effects of periodontal treatment in patients with diabetes. In this case, different outcomes should be assessed, including clinical, financial as well as patient-reported outcomes. This could confirm the results of the current and previous studies regarding the different outcomes as a result of periodontal treatment. Furthermore, such a study would allow for assessment of this association in different severities of periodontitis as well as differences in HbA1c levels. This would provide more information about the actual relationship between periodontitis and diabetes. Possibly, this information could justify insurance coverage for periodontal treatment for individuals with diabetes, as well as investments in adequate training for physicians and dentists on integration and coordination of healthcare.

In summary, periodontal treatment in individuals with assumed diabetes may potentially reduce diabetes-related healthcare costs, in addition to improving HbA1c levels. These findings underscore the potential importance of periodontal treatment in individuals with diabetes. They may support initiatives to promote additional attention to the periodontal status of individuals with diabetes and to raise more awareness for the benefits of periodontal treatment. Future research is needed to assess the exact financial benefits of adequate periodontal treatment in patients with diabetes.
