# Supplementary material for: Effect of periodontal treatment on diabetes-related healthcare costs: a retrospective study
Source: BMJ Open Diabetes Res Care. 2020 Oct 23;8(1):e001666. doi: 10.1136/bmjdrc-2020-001666 (PMC7590362; doi:10.1136/bmjdrc-2020-001666)
Supplement: Supplementary data [file bmjdrc-2020-001666supp001.pdf]

Online-Only Supplement to 'The effect of periodontal treatment on diabetes-related healthcare costs: A retrospective study'

Content

eTable 1. Periodontal treatment classification and reimbursement codes 2
eTable 2. Diabetes healthcare costs classification and diagnosis, treatment and anatomical therapeutic chemical codes 3
eTable 3. Sensitivity analyses for fixed effect panel regression models 4
eTable 4. Fixed effect regression model in subsamples of study population 5

eTable 1. Periodontal treatment classification and reimbursement codes

Category	Codes
Initial periodontal therapy	T21; T22; TI99
Periodontal surgery	4024; 4025; 4027; N311; N315(Z); N411(A/Z); N511(A/Z); N515(A/Z); N551; N611; N615(A); T57; T70; T71; T72; T76; T80; T81; T82; T83; T84; T85; T86; T87; T88
Supportive periodontal therapy (short visit)	T51; T52
Supportive periodontal therapy (normal visit)	N111; T53; T54; T75; T99; TB99; TH99; TN99; W112
Supportive periodontal therapy (long visit)	T33; T55; T56; W114
Treatment of complications of periodontitis, such as periodontal abscess	N811(Z); T94
Additional technical costs for materials, such as periodontal splint	T00; T95

eTable 2. Diabetes healthcare costs classification and diagnosis, treatment and anatomical therapeutic chemical codes

Category	Codes
	Diagnosis and Treatment codes (DBC)
Diagnosis	040201001; 040201004; 040201012; 040201015
Treatment	040201008; 040201010; 040201013; 140301034; 140301055; 140301062
Insulin pump	040201009; 040201011; 040201014; 040201016; 040201020; 040201021; 140301015; 140301016
Hospitalization	040201006; 040201007; 040201019; 140301014; 140301035; 140301036; 140301038; 140301039; 140301080
Complication eye	079799003; 079799006; 079799007; 079799012; 079799022; 079799023; 079799024; 079799025; 079799031; 079799032; 079799033; 079799034; 079799036; 079799037; 079799038; 079799041; 079799045; 079799048
Complication foot	099699007; 099699018; 099699019; 099699020; 099699021; 099699022; 099699023; 099699034; 099699035; 099699036; 099699058; 099699059; 099699060; 099699063; 099699064; 099699076; 099699083
	Anatomical Therapeutic Chemical codes (ATC)
Only metformin	A10BA02
Other oral glucose lowering drugs	A10B excluding A10BA02
Insulin	A10A
	Tool codes
HbA1c Test strips	F10

eTable 3. Sensitivity analyses for fixed effect panel regression models

Model	N	Coefficient	95%CI	p-value
**Sensitivity 1: Categories of diabetes healthcare costs**				
Total diabetes medication-related costs	41,598	€4.92	€3.93; €5.91	<.001
Total diabetes non-medication related costs	41,598	€-16.95	€-20.53; €-13.37	<.001
**Sensitivity 2: Medication groups in 2012**				
Only metformin	14,534	€7.38	€4.38; €10.37	<.001
Other oral glucose-lowering drugs	13,254	€13.56	€8.51; €18.61	<.001
Insulin	13,810	€-58.09	€-67.83; €-48.35	<.001
**Sensitivity 3: Time lag**				
6 month time lag	41,598	€-11.96	€-15.60; €-8.32	<.001
1 year time lag	41,598	€-12.01	€-16.52; €-9.49	<.001

CI: confidence intervals

eTable 4. Fixed effect regression model in subsamples of study population

Subsample	N	Coefficient	95%CI	p-value
**Age**				
≤65 years old	26,415	€-9.53	€14.43; €-4.63	<.001
>65 years	15,183	€-17.79	€-23.32; €-12.27	<.001
**Gender**				
Male	22,605	€-8.49	€-13.73; €-3.24	0.002
Female	18,992	€-16.89	€-22.16; €-11.61	<.001
**SES**				
Very low – low	19,618	€-12.64	€-17.77; €-7.52	<.001
Neutral – very high	21,562	€-11.58	€-17.07; €-6.09	<.001

CI: confidence intervals; SES: Socioeconomic status
